# A protocol for setting‐up robust hydrophobic interaction chromatography targeting the analysis of intact proteins and monoclonal antibodies

**DOI:** 10.1002/ansa.202200058

**Published:** 2022-12-24

**Authors:** Raphael Ewonde Ewonde, Nico Lingg, Daniel Eßer, Sebastiaan Eeltink

**Affiliations:** ^1^ Department of Chemical Engineering Vrije Universiteit Brussel (VUB) Brussels Belgium; ^2^ Department of Biotechnology Institute of Bioprocess Science and Engineering University of Natural Resources and Life Sciences Vienna Austria; ^3^ YMC Europe GmbH Dinslaken Germany

**Keywords:** biopharmaceuticals, HPLC method development, native liquid chromatography, protein profiling

## Abstract

Hydrophobic interaction chromatography (HIC) is a chromatographic technique that mainly targets the separation of biomolecules (intact proteins, monoclonal antibodies, etc.) based on the difference in surface hydrophobicity while applying non‐denaturing conditions. This protocol paper provides guidelines for setting‐up robust HIC analysis and considers the instrument configuration, mobile‐phase and sample preparation, as well as chromatographic conditions and settings. The separation of a mixture of intact proteins and monoclonal antibodies is demonstrated by applying conventional HIC conditions, that is, using a mildly hydrophobic (C_4_) stationary phase in combination with an inverse ammonium sulphate gradient dissolved in aqueous phosphate buffer. The effect of sample‐preparation conditions on sample breakthroughs is presented. Finally, good run‐to‐run repeatability (relative standard deviation < 2%) is demonstrated for five different columns obtained from three different column lots, considering chromatographic retention, peak width, peak area and column pressure.

## 1 INTRODUCTION TO HYDROPHOBIC INTERACTION CHROMATOGRAPHY

In 1948, Tiselius reported on a separation experiment where proteins adsorb to a stationary phase in the presence of a salt concentration that is only slightly lower concentration than is required for their precipitation.^1^ It was observed that adsorption was reversible and could be mediated by the salt concentration. Tiselius referred to this technique as ‘*adsorption separation by salting out’*, which was later renamed by Hjertén to hydrophobic interaction chromatography (HIC).^2^ Similar to reversed‐phase liquid chromatography, HIC separates biomacromolecules based on their hydrophobicity. However, as non‐denaturing conditions are applied, the protein 3D conformation and hence biological activity is (largely) maintained.^3^

**FIGURE 1:**
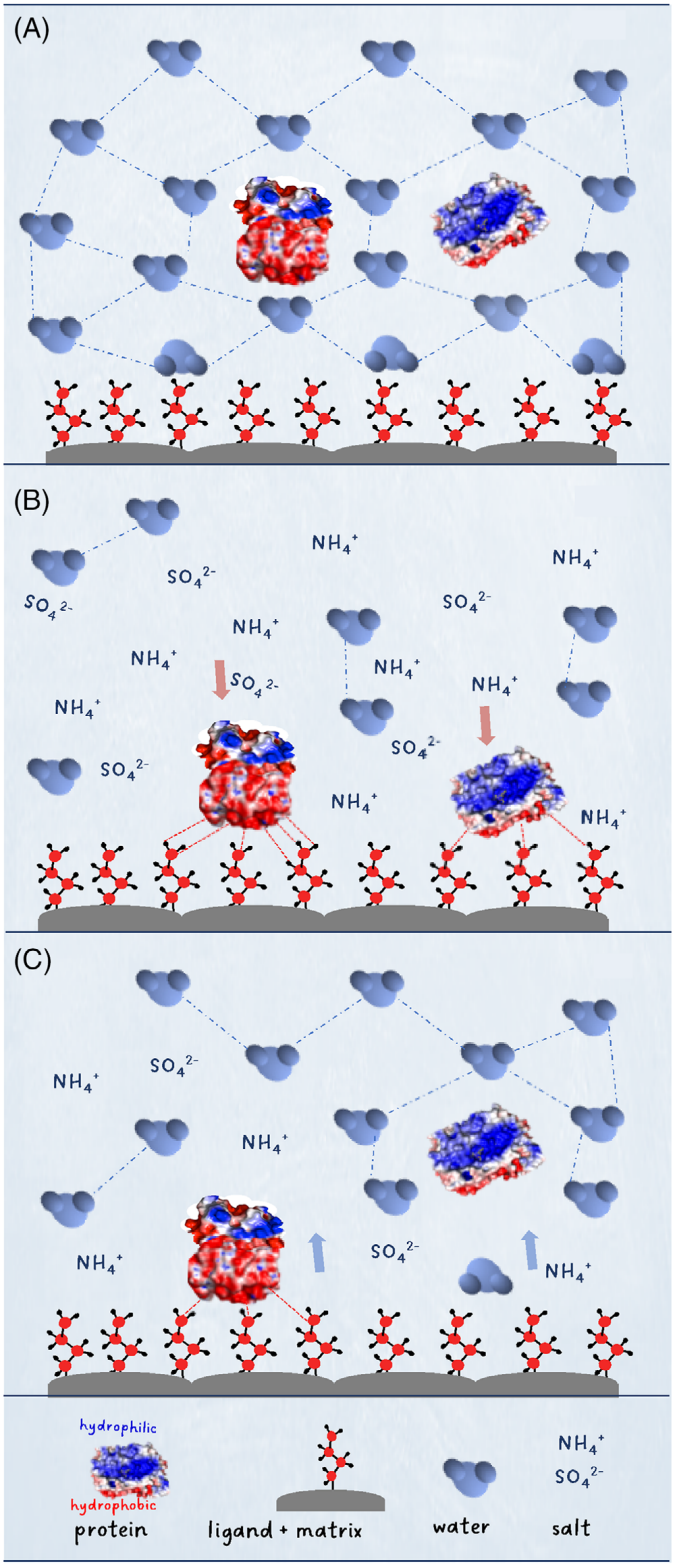
Retention mechanism for hydrophobic interaction chromatography. (A) At aqueous conditions, ordered‐water layers are formed shielding the protein and stationary phase surface. (B) At high salt concentrations the ordered‐water layers are disrupted, exposing hydrophobic surfaces. Proteins and stationary‐phase ligands associate via hydrophobic interactions driven by the increase in entropy. (C) Hydrophobic interactions gradually weaken when decreasing the ionic strength of the mobile phase and proteins elute sequentially according to their surface hydrophobicity.

Hydrophobic interactions between analytes and the mildly hydrophobic stationary phase in an aqueous environment are driven by the change in entropy. In an aqueous environment, the stationary‐phase surface and proteins containing both hydrophilic (displayed in blue) and hydrophobic (displayed in red) moieties are shielded by ordered water layers, see Figure 1A. The high ionic strength applied at the start of the gradient disrupts the ordered water layers in the immediate vicinity of the protein and the stationary‐phase ligands, as salt ions are preferentially hydrated in an aqueous environment, see Figure 1B. As the entropy (Δ*S*) increases, while the enthalpy (Δ*H*) and temperature (*T*) remain constant, hydrophobic interactions are promoted by negative Gibbs free energy (Δ*G*),^4^

(1) ΔG=ΔH−T·ΔS

Proteins with more hydrophobic moieties will undergo stronger interactions with the hydrophobic stationary‐phase ligands than those with more hydrophilic moieties and hence display more retention. Upon decreasing the salt concentration, the hydrophobic interactions gradually weaken as entropy is no longer a driven force. The ordered water layers are reformed, and analytes will elute in increasing order of surface hydrophobicity, see Figure 1C.

Separations based on hydrophobic interaction mediated by salt are applicable to a wide range of analytes, including some low molecular‐weight analytes (<2000 Da) and (bio‐)macromolecules. Considering the HIC analysis of low‐molecular weight analytes (aromatic alcohol homologues, dansylamino acid) studies are merely limited to retention‐time profiling while varying column chemistry and temperature.^5^, ^6^ Proof‐of‐concept to separate peptides and oligonucleotides with HIC has been demonstrated, albeit the achieved resolving power is currently lower compared to reversed‐phase liquid chromatography (RPLC).^7^, ^8^ HIC has been extensively used for the analysis of biomacromolecules such as globular proteins, including proteoforms,^9^, ^10^, ^11^ recombinant monoclonal antibodies (mAbs),^12^ and antibody‐drug conjugates (ADCs).^13^ Critical quality attributes of ADCs that have successfully been analyzed, include the average drug‐to‐antibody ratio,^14^ and drug‐load distribution.^15^ Recently, new applicability areas have been explored, including the HIC purification of viruses,^16^ and the analysis of plasmids^17^ and exosomes.^18^

## 2 DEVELOPMENT OF THE PROTOCOL

**FIGURE 2:**
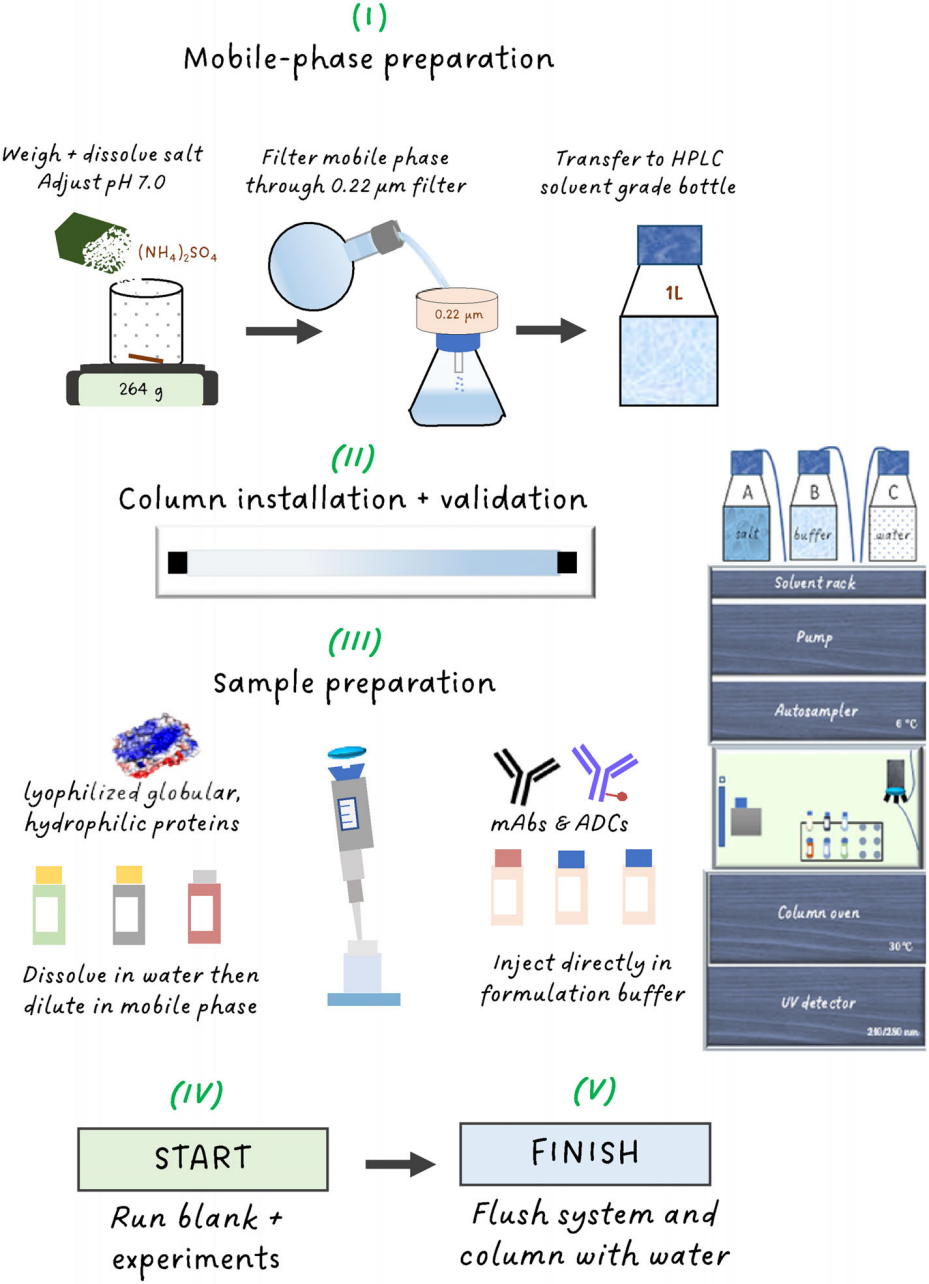
Protocol scheme for setting‐up conventional analytical hydrophobic interaction chromatography.

Although HIC has been successfully applied for the analysis of a range of biomolecules, method robustness is still considered an issue. HIC involves the use of high salt concentrations and improper system care eventually leads to salt precipitation in the fluidics. Consequently, this leads to unrepeatable LC performance and ultimately irreversible column and system damage. This protocol focuses on establishing robust HIC conditions, targeting the analysis of intact proteins and monoclonal antibodies. Figure 2 shows the protocol scheme for setting‐up robust HIC. Guidelines for mobile‐phase and sample preparation, system configuration and conditioning are provided below. The effect of solvent composition on sample breakthrough is demonstrated. HIC analyses were performed using 4.6 × 100 mm (internal diameter [i.d.] × L) columns packed with 2.3 µm nonporous particles functionalized with butyl moieties and applying conventional elution conditions defined as an inverse linear ammonium sulfate gradient dissolved in 50 mM phosphate buffer (pH 7.0). Finally, run‐to‐run and column‐to‐column repeatability is demonstrated considering retention time, peak width and column pressure for three different column batches.

## 3 CHEMICALS AND MATERIALS

Ammonium sulfate (≥99.0%), disodium hydrogen phosphate (≥99.0%), sodium dihydrogen phosphate (≥99.0%), sodium hydroxide (high‐performance liquid chromatography [HPLC] grade, 50.0%), sodium azide, myoglobin (equine heart), ribonuclease A (bovine), lysozyme (chicken egg white), trypsinogen (bovine pancreas), carbonic anhydrase (bovine erythrocytes) and α‐chymotrypsinogen A (bovine pancreas) were purchased from Sigma‐Aldrich (Bornem, Belgium). Monoclonal antibodies (adalimumab, trastuzumab and infliximab) were provided by BOKU (Vienna, Austria). HPLC‐grade deionized water was produced in‐house using a Milli‐Q water purification system (Millipore, Molsheim, France).

4.6 mm i.d. × 100 mm columns packed with 2.3 µm (BioPro HIC HT) non‐porous polymethacrylate particles functionalized with a butyl chemistry were obtained from (YMC Europe, Dinslaken, Germany).

## 4 HPLC INSTRUMENTATION, CONFIGURATION AND MAINTENANCE

HIC experiments were conducted using an Ultimate 3000 BioRS system (Thermo Fisher Scientific, Germering, Germany) composed of a solvent rack with integrated membrane degassers, a ternary low‐pressure gradient pump, a well‐plate autosampler enabling in‐line split‐loop (flow‐through needle) injections, a forced‐air column oven and a diode‐array UV detector. 180 µm i.d. tubing was used to connect the pump to the injector. To maintain high separation efficiency the system was configured with 100 µm i.d. tubing between the injector and the column, and the column and the detector. The injection volume was set at 5 µL. The temperature of the well‐plate sampler was set at 6°C and the column oven was maintained at 30°C. The flow rate was set at either 0.5 or 1 mL/min, see details in the figure captions. A 2.5 µL UV flow cell was used, and data was recorded at 210 and 280 nm, applying 5 Hz data collection rate and 1 s response time. Chromeleon software (version 7.2.10) was used for system control and data management.

Mobile phase A consisted of ammonium sulfate dissolved in phosphate buffer, mobile phase B consisted of phosphate buffer and mobile phase C consisted of deionized water. After installing the HIC column, the column was equilibrated with water to remove the storage solution comprising organic solvent and to prevent salt precipitation. Hereafter, the system was equilibrated first with buffer (mobile phase B) for 30 min, followed by mobile phase A for 10 min applying a flow rate of 0.5 mL/min, which corresponds to 30 and 10 column volumes, respectively. After analysis, the system and column were flushed with deionized water to remove residual salt, and the column was stored in the storage solution prescribed by the manufacturer. When the system was temporarily not in use, it was flushed with deionized water spiked with 0.05% sodium azide at a flow rate of 0.08 ml/min to prevent salt precipitation and bacteria growth in the fluidics.

Note that:

- Monitoring of the system pressure is important as an increase in system pressure may indicate salt precipitation. If the pressure increases significantly (>20 bar) it is recommended to flush the system with deionized water.
- Periodic (bi‐monthly) system maintenance is advised, which includes cleaning the filter frit and flushing the system with warm water (70°C; disconnected flow cell).
- In case a significant increase in system pressure is obtained, check membrane degassers and remove salt crystals within the membrane, if any. Also, open the pump head and rinse and sonicate plungers and seals with deionized water.

## 5 MOBILE‐PHASE PREPARATION

Conventional HIC is performed applying a linear inverse ammonium sulfate gradient. Mobile phase A was composed of 2.3 M ammonium sulphate dissolved in 50 mM phosphate buffer pH 7.0. Mobile phase B was 50 mM aqueous phosphate buffer pH 7.0. As the addition of ions used contributes to the overall retention, it is recommended to apply the Henderson‐Hasselbalch buffer formula and calculate the exact amounts of salt needed to establish the required buffer capacity and pH, while adding a minimum of sodium hydroxide in the case of a phosphate buffer to reach, for example, pH = 7.0. The recommended mobile‐phase preparation is as follows:

- Add 600 ml of deionized water in a 1 L beaker and stir continuously while dissolving the weighted salts, that is, 303.60 g ammonium sulfate, 2.71 g of dibasic sodium phosphate, and 3.71 g of monobasic sodium phosphate for mobile phase A, and 2.71 g of dibasic sodium phosphate and 3.71 g of monobasic sodium phosphate for mobile phase B and add water to reach 950 ml.
- Monitor the pH with a calibrated pH meter and add 2 M aqueous NaOH solution with a dropping pipette until a pH of 7.0 is reached.
- Transfer the solution to a 1 L volumetric flask and make up to 1.0 L with deionized water.
- Filter the solutions through a 0.22 µm filter using a water jet pump, to remove microparticles and any salt clusters. Then transfer the solution to a 1 L HPLC grade solvent bottle.

Note that:

- The purity and hence color of the salt (less or more yellowish) may vary significantly between manufactures. The use of yellow ammonium sulfate impacts the UV baseline and reduces the linear detection range.
- Filtering the mobile phase reduces the pressure fluctuation and column deterioration, and hence will advance the system robustness.
- Prepared mobile phases have limited shelf time depending on the storage temperature. Typically, mobile phases are replaced every week.

## 6 SAMPLE PREPARATION

In the ideal case, the sample is dissolved in the starting mobile phase to prevent sample break‐through, defined as the elution of analyte at the time of an unretained analyte (*t_0_* time). Depending on the salt concentration and protein, precipitation may occur. To overcome this problem, the protein stock solution can be prepared in water and target salt concentration can be reached by dilution with ammonium sulfate solution (mobile phase A). The procedure is as follows:

- Weigh the appropriate mass of lyophilized sample and transfer into a clean Eppendorf tube and add deionized water to reach the protein stock concentration. Mix gently by vortexing. Protein stock solutions can be stored at ‐4°C in the freezer for later use.
- Dilute stock sample solution with mobile phase A to the desired concentration. It is recommended to maintain the ammonium sulphate concentration ≥ 50% of the starting salt concentration to achieve on‐column focusing and to prevent sample break through (see discussion below).

Note that,

- Crude extracts (e.g. cell lysate) can be filtered via 0.22 µm filter membrane to remove solid microparticles.
- How long the stock sample can be stored at ‐4°C is protein specific. A change in chromatographic peak profile of the analyzed proteins can be observed after prolonged sample storage (‐4°C) if the protein stock solution is prepared in a concentrated salt solution instead of water.

## 7 METHOD‐DEVELOPMENT GUIDELINES

**FIGURE 3:**
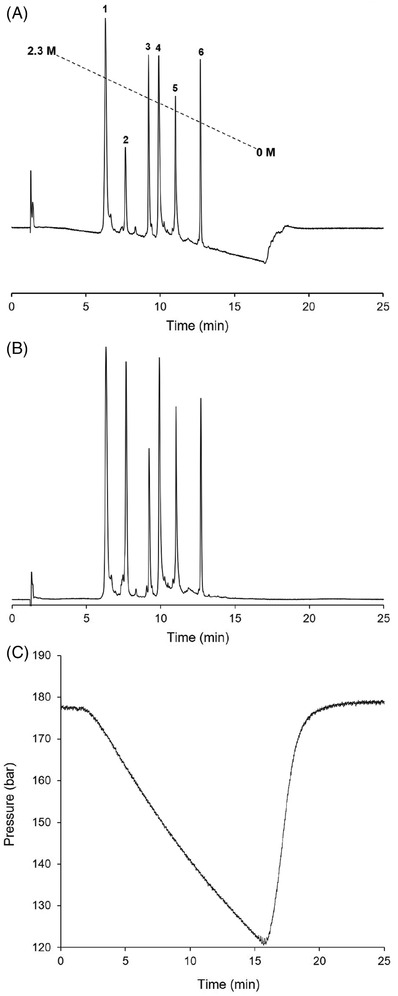
Optimized separation of a mixture of globular proteins, before (A) and after (B) blank subtraction, and the corresponding pressure profile (C). Experimental conditions: the gradient duration was 15 min and the gradient span Δ*c* was 2.3–0 M ammonium sulfate in 50 mM phosphate buffer at pH = 7.0. Flow rate = 0.5 mL/min and the column oven was set at 30°C. Peak identification: (1) myoglobin, (2) ribonuclease A, (3) lysozyme, (4) carbonic anhydrase, (5) trypsinogen and (6) α‐chymotrypsinogen A.

Figure 3 shows typical chromatograms of the separations of intact proteins that cover a wide range in hydrophobicity range applying conventional HIC conditions, that is, using a column packed with non‐porous C_4_ particles and applying a 15 min inverse aqueous ammonium sulfate gradient dissolved in 50 mM phosphate buffer pH 7.0. Figure 3A shows the resulting chromatogram, whereas Figure 3B shows the same separation after blank subtraction. The corresponding pressure trace is depicted in Figure 3C. In this case, the mobile phase was filtered prior to use following this protocol. When omitting mobile‐phase filtration, significant pressure fluctuation is obtained, which increases over time, negatively affecting retention‐time repeatability. As such, mobile‐phase filtration is strongly recommended.

Most column manufacturers currently target the analysis of mAbs using C_4_ column chemistry. The differences in surface coverage of C_4_ chains, and therefore column hydrophobicity, affect the retention capacity, and it may be necessary to adjust the salt concentration accordingly. When analyzing highly hydrophobic macromolecules, such as some antibody‐drug conjugates, either the use of a slightly more hydrophilic column can be considered, or one can use a less kosmotropic salt system, such as sodium chloride. A typical sodium chloride gradient starts at 4 M of salt. A change in column chemistry and/or salt type may result in a change in chromatographic selectivity. The addition of organic solvents to the HIC mobile phase has also been reported as an alternative approach to reduce the strength of the hydrophobic interaction.^19^ It is important to realize that this may affect the 3D conformation. Our group demonstrated that the addition of only 2.5% (v/v) of IPA to the HIC mobile phase already leads to denaturation of *α*‐ lactalbumin.^20^ This effect is protein specific. The addition of significant amounts of organic modifier (up to 50% [v/v]) to HIC mobile‐phase systems have been reported.^21^ It is questionable whether the 3D conformation and corresponding biological activity can be maintained under these conditions, and this may strongly depend on type of sample analyzed (oligonucleotides, plasmids, proteins). The elution of analytes is in this case governed by both the salt content (HIC interaction) and the amount of organic solvent (RPLC partitioning) in the mobile phase.

**FIGURE 4:**
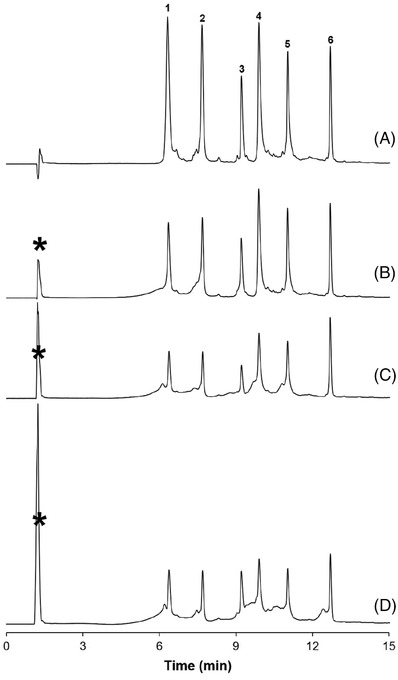
Effect of the salt concentration in the sample solution on chromatographic retention, peak profiles, and sample breakthrough (marked with an asterisk). The ammonium sulfate concentration in the sample solutions were adjusted to (A) 1.7 M, (B) 1.2 M, (C) 0.5 M and (D) 0 M. Experimental conditions and peak identification is similar as in Figure 3. The asterisk represents the sample breakthrough peak.

A large difference in the elution strength between the mobile‐phase starting conditions and the sample may give rise to ‘*sample breakthrough*’, which is defined as the early elution of analytes, close to the column dead time which occurs when analytes experience (almost) no retention and hence is dissolved in a strong solvent. Figure 4 shows the separation of intact proteins while varying the ionic strength of the sample solvent. When applying a sample solution of low ionic strength, the peak area of the analytes is lower than when applying a sample solution of high ionic strength. Moreover, a breakthrough peak, marked with an asterisk, is observed, that contains proteinaceous material. Water is considered a strong solvent in HIC shielding the hydrophobic sites of the protein and preventing their interaction with the stationary phase. The mismatch between sample solvent and the mobile‐phase composition compromises the accurate quantification. By increasing the ionic strength in the sample solution this problem can be circumvented.

## 8 ROBUSTNESS AND COLUMN REPEATABILITY

**FIGURE 5:**
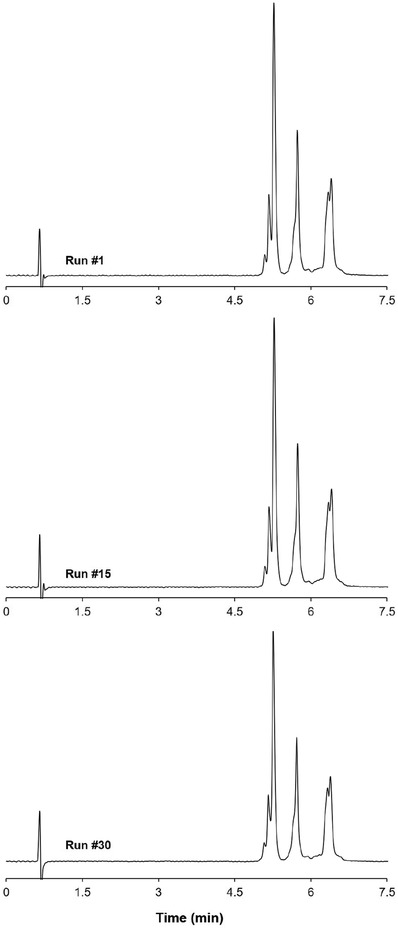
Overlay of chromatograms of a mixture of monoclonal antibodies (mAbs) demonstrating run‐to‐run repeatability. Experimental conditions: the gradient duration was 7.5 min and the gradient span Δ*c* was 1.7–0 M ammonium sulfate in 50 mM phosphate buffer at pH = 7.0. Flow rate = 1 ml/min and the column oven was set at 30°C. Peak identification: (1) adalimumab, (2) trastuzumab and (3) infliximab.

**FIGURE 6:**
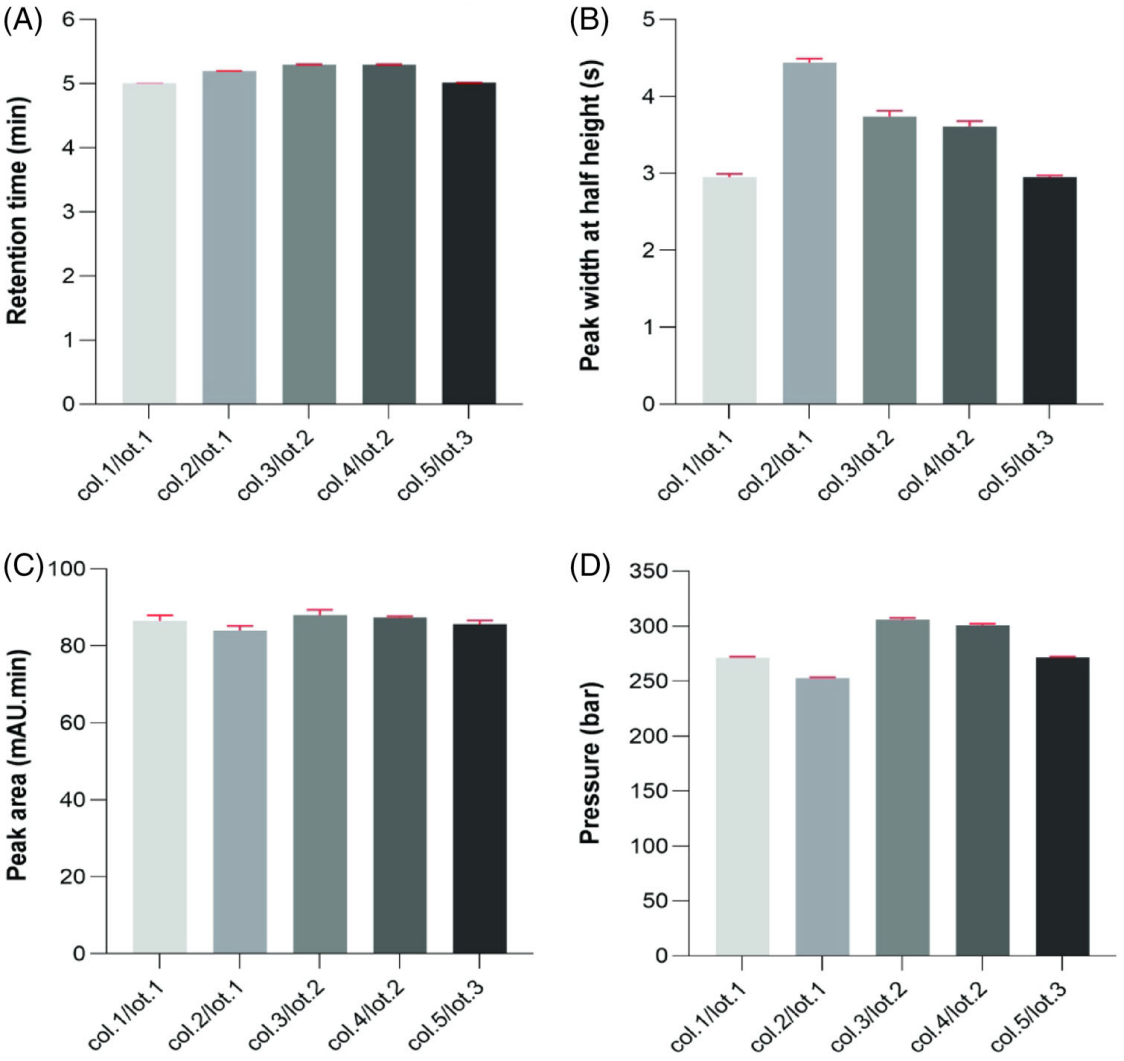
Column repeatability for different columns from three different lot numbers. Retention time, peak width and half height and peak area were determined for adalimumab. Experimental conditions are similar as stated in Figure 5.

Column robustness was assessed by measuring retention time, peak width and peak area for 30 consecutive injections applying a single column and 7.5 min ammonium sulfate gradients. Figure 5 shows the resulting overlay of blank‐subtracted chromatograms (run #1, #15 and #30) of the separation of adalimumab, trastuzumab and infliximab. Statistical identical variances of retention times, peak width and peak area for adalimumab were confirmed by Fisher tests of variances, see Table 1. In a next step, the repeatability performance of five different HIC columns obtained from three different column lots was assessed following ICH guidelines, which involves comparison of six subsequent analysis runs on each column. Figure 6 depicts, for each of the columns, the corresponding mean and statistical deviation for retention time, peak width recorded at half height, peak area and column pressure recorded at the gradient start. The relative standard deviations (RSDs) for all parameters remain small and do not exceed 2% and hence remain within the threshold set by the ICH, see Table 2 for quantitative data.

**TABLE 1:** Run‐to‐run repeatability obtained for adalimumab in gradient hydrophobic interaction chromatography (HIC) mode.

***n* = 30**	**Retention time (min)**	**Peak width at half height (s)**	**Peak area (mAU min)**
Mean	5.272	3.26	77.06
Inferior/superior limit at 5% risk	5.270‐5.277	3.24‐3.330	75.97‐77.15
Variance	0.000	0.00	3.45
RSD (%)	0.119	1.64	2.41

**TABLE 2:** Column‐to‐column repeatability data obtained for five different hydrophobic interaction chromatography (HIC) columns originating from three different lots.

**Column no./lot no**.		**Retention time (min)**	**Peak width at half height (s)**	**Peak area (mAU min)**	**Pressure (bar)**
1/1	Mean	5.008	2.95	86.55	271.3
	SD	0.001	0.04	1.26	0.7
	RSD	0.028	1.40	1.46	0.3
2/1	Mean	5.199	4.44	84.02	252.7
	SD	0.002	0.05	1.05	0.9
	RSD	0.029	1.10	1.25	0.4
3/2	Mean	5.016	2.95	85.70	271.8
	SD	0.002	0.02	0.83	0.4
	RSD	0.038	0.76	0.96	0.1
4/2	Mean	5.297	3.74	88.01	306.0
	SD	0.003	0.07	1.25	1.5
	RSD	0.055	1.77	1.42	0.5
5/3	Mean	5.300	3.61	87.41	300.8
	SD	0.002	0.06	0.25	1.3
	RSD	0.039	1.77	0.29	0.4

## 9 CONCLUDING REMARKS

The aqueous mobile phase and neutral pH generally employed in HIC offers the possibility to maintain the 3D structure (and function) of a protein. Although the HIC conditions are considered to be mild for proteins, the high salt concentration is problematic for the HPLC instrumentation. Here, we have established a step‐by‐step procedure for setting up HIC for the analysis of intact proteins and monoclonal antibodies leading to robust performance. To prevent sample breakthrough, the salt concentration of the sample solvent must ideally match the gradient start composition. With the conditions established here, good run‐to‐run repeatability on five different columns (RSD for retention time, peak width, peak area and pressure < 2%) was achieved allowing to establish HIC for routine analysis in QC labs.

## AUTHOR CONTRIBUTIONS

**Raphael Ewonde Ewonde**: Investigation and writing – original draft. **Nico Lingg**: Sample preparation and reviewing. **Daniel Eβer**: Conceptualization and reviewing. **Sebastiaan Eeltink**: Funding acquisition, conceptualization, writing, review & editing and supervision.

## CONFLICT OF INTEREST

VUB and BOKU authors declare that they have no conflict in interest. Sebastiaan Eeltink is Editor‐in‐Chief of Analytical Science Advances. Daniel Eβer is an employee of YMC Europe.

## Data Availability

The data that support the findings of this study are available from the corresponding author upon reasonable request.
